# The Book World of Medicine and Science

**Published:** 1911-07-29

**Authors:** 


### THE BOOK WORLD OF MEDICINE AND SCIENCE.

**E. Merck's Annual Report of Recent Advances in Therapeutics.** Vol. xxiii. 1909. Darmstadt: E. Merck. 1910. Pp. 381. Price 1s. 6d. net.)

The appearance of a new volume of this well-known annual calls for little comment except to say that in it the author has displayed the same thoroughness which has characterised his former productions. No fewer than eighty-four pages of the present book are devoted to an historical and practical review of serum-therapy and bacterio-therapeutic preparations. We noticed, however, one slip on page 2, where it is stated that vaccination was introduced into England by Lady Montague in 1721, whereas that lady is, of course, famous for having introduced the practice of inoculation into this country. Vaccination properly so-called was really introduced by Jenner in 1798, as the author states in a later paragraph. Some 263 pages are then given over to a very thorough review of the therapeutic preparations and drugs introduced during the year. A limited number of copies of the book will be sent post free to medical practitioners on application to the firm's London office, 16 Jewry Street, E.C.; but the book will also be obtainable through booksellers at the price stated above.

**Unsoundness of Mind.** By T. S. Clouston, M.D., LL.D., F.R.S.E. (London: Methuen and Co., Ltd. 1911. Price 7s. 6d. net.)

It is the author's intention to present to the intelligent layman the latest views and discoveries with regard to those mental diseases, perversions, and defects which affect mankind and influence human conduct. We may say at once that in this he has been successful, for he has produced a work which not only the layman but the medical practitioner will read with profit. While the text is free from every unnecessary technicality of language, the subject is treated in an orderly, intelligible fashion, such as one would expect from a writer of the author's acknowledged eminence in his own specialty. The work should be of the utmost value to those who, by reason of their position as legislators or guardians, have the care of the insane, for it is full of practical information resulting from ripe experience. The appearance of a popular work of this nature cannot fail to reassure the public that the profession has no desire to treat the insane in a spirit of secrecy, such as a number of modern journals would seem to imply.

